# Building a more engaged scientist from the bottom up: The impact of public engagement training on undergraduate students

**DOI:** 10.1371/journal.pone.0302671

**Published:** 2024-04-30

**Authors:** Nikole D. Patson, Laura Wagner

**Affiliations:** Department of Psychology, Ohio State University, Columbus, OH, United State of America; Institute of Medical Biochemistry Leopoldo de Meis (IBqM) - Federal University of Rio de Janeiro (UFRJ), BRAZIL

## Abstract

Engaging with the public is increasingly seen as an important role of scientists. Despite that, few opportunities exist for undergraduate students to receive training in engaging with the public about science. Thus, little is known about the impact of such training on students. The goal of the current study was to investigate the impact of public engagement training on participants in a summer program for undergraduates that provides training in both research and engagement activities. The results of our interviews suggest that providing opportunities for undergraduates to engage with the public (1) has many personal, academic, and career benefits for students; (2) increases participants’ interest in public engagement; and (3) may contribute to helping students develop and maintain an identity as scientists. Importantly, students from minoritized racial groups may be even more impacted by this experience. These data suggest that early experiences with public engagement may not only be an important way to increase the number of publicly engaged scientists but may also broaden participation in science.

## Introduction

Beginning in the 1990’s research funders and academic institutions began putting greater emphasis on scientists participating in public engagement [1]. That is, talking with the general public about science. For example, in the mid-1990s, the National Science Foundation (NSF) implemented a Broader Impacts Criterion as part of the application process for research funding [2]. Applicants for funding are required to provide a description of how a proposed research project will affect the broader society via teaching, inclusion of underrepresented groups, the creation of community relationships for outreach, public discussion of research findings, and the general social benefits of the project (See http://www.nsf.gov/pubs/gpg/broaderimpacts.pdf Accessed 4/11/22). Proposal reviewers are instructed to consider the broader impacts equally as important as the scientific merit of any proposal, emphasizing the importance of public engagement to the NSF.

Although communicating with the public is increasingly recognized as a responsibility of scientists [3,4], the shift towards greater public engagement by scientists has been slow. One factor that may explain the slowness of progress could be the fact that training in public engagement is largely ignored in science education [5]. The lack of training opportunities may be one reason why so many academic scientists feel that they and their peers do not have the necessary skills to engage the public [6–9]. While some training opportunities do exist, they rarely target undergraduate students [10]. If public engagement training begins early in a scientist’s career, this may increase the number of scientists who do this work. However, given the lack of public engagement training opportunities for undergraduate students, we know very little about the potential impact of this training [cf., 10]. The current study investigated how public engagement training might impact undergraduates’ views of science and their own role as scientists.

### Benefits of participating in public engagement training

Public engagement with science can be considered “mutually beneficial” for scientists and the public. The benefit to the public is clear: They gain access to current scientific thinking and, feel inspired, and learn about different STEM careers [11]. Scientists who engage with the public also report benefits [12–16]. For example, Andrews et al., (2005) found that scientists reported feeling a sense of contributing to the greater good as a top benefit for participating in public engagement. As one researcher put it, public engagement “when done correctly-is one of the most valuable contributions a professional scientist can make” [12, p. 285].

Students who engage with the public report additional gains. Graduate students report career-related skills such as communication, teamwork, and collaboration [13–16]. Graduate students also report gaining a better understanding of career options and clarifying their career interests through participating in public engagement [14,15,17]. Across studies, graduate students experienced gains in science content knowledge and improved their science teaching skills [14,16,18]. Importantly, they also report a greater understanding of the importance of university–K-12 engagement [14,15,19].

To our knowledge, one study has investigated undergraduate students’ perceived gains from participating in science engagement with K-12 students [10]. Carpenter (2015) interviewed science education majors who had participated in one of several different types of science engagement programs located in a K-12 setting. The participants in the study reported career, academic, and personal gains. They also recognized that understanding students, the nature of science and scientific practices, active learning, and student interest are important for teaching and learning science. Importantly, the students participating in this study were training to be science educators, not laboratory scientists. While science engagement with K-12 students is clearly aligned with the goals of education, it is not traditionally taught as a part of the scientific process used by scientists [e.g., 20]. Thus, undergraduate students who are training to be research scientists may not have the same reaction to science engagement as students studying to be educators. Thus, it is important to understand how public engagement training impacts future scientists at the early stages of their education.

### Barriers to engaging with the public for academic scientists

Academic scientists report several institutional and personal factors that limit their ability to engage with the public. A lack of time and lack of institutional support emerge as two of the most important barriers [6,12,21]. Academic scientists do not feel they can devote time to public engagement due to their other university duties [6,12]. Faculty report receiving little institutional support for setting up public engagement initiatives and raise concerns about how these efforts would (not) be rewarded in annual review and tenure processes [12]. These findings make clear that institutions of higher education largely see engaging with the public as outside of the job duties of academic scientists.

Additionally, some scientists report lacking the necessary skills to share their research more broadly, perceiving themselves as having poor social skills, and having little confidence in their ability to engage the public [6,8,9]. This highlights the need for early training opportunities to help build scientists’ confidence in their ability to engage with the public.

### Public engagement training from the bottom up

One factor that may contribute to perceptions that public engagement is secondary to conducting experiments and publishing academic articles is how scientists are trained at the earliest stages of their careers. It is widely understood that participating in undergraduate research is valuable for students and there has been a large push to increase research experiences for undergraduate students [22,23]. Indeed, these experiences are valuable: Students gain technical and interpersonal skills [24], analytical, logic, synthesis, and independent learning skills [25], while improving their ability to gain entrance into competitive graduate programs [26]. However, efforts to train students in science rarely train them to engage with the public. Lopatto (2003) interviewed faculty and undergraduate students about their views of the essential features of a successful undergraduate research experience. Faculty and students had similar ideas as to what makes a good research experience, but important to our goals, neither faculty nor students discussed public engagement as a critical component of undergraduate research. Given the absence of attention to public engagement, it is understandable that few opportunities exist for undergraduates to engage with the public.

The lack of opportunities for undergraduate students to engage with the public not only means scientists don’t get access to this training early on in their careers, but it may also contribute to why scientists (and institutions) do not view public engagement as a fundamental part of a scientist’s job. We take this view that it is fundamental, based on an experiential learning perspective. Experiential learning considers experience the central factor in learning. Experiential learning experiences offer learners the opportunity to engage in authentic experiences which help them build knowledge, understanding, and skills [27,28]. Undergraduate research experiences not only teach students how to do research, but they also teach students about the nature of a scientist’s job. By ignoring public engagement, students are being taught that engagement is not a central part of being a scientist.

In fact, the literature shows that when graduate students seek out public engagement opportunities, they face several challenges. Graduate students report feeling a loss of standing in their research groups, setbacks in their own research, and lack of support from advisors [14,17,18]. These reported challenges reflect the typical academic attitude that a scientist’s primary job is to do research. One possible benefit of training undergraduate students in public engagement is that their early experiences of science would include public engagement and as a result they may adopt the attitude that public engagement is a critical component of a scientist’s work.

### Public engagement training may broaden participation in science

Including public engagement training for students may have additional benefits. Specifically, public engagement has the potential to broaden interest and participation in science. One outcome of participating in undergraduate research is that students often learn that they are not interested in pursuing academic research, and this decreased interest in pursuing a science career is not equally distributed. Kardash, Wallace, and Blockus (2008) found that women, more than men, were likely to report that participating in undergraduate research decreased their interest in pursuing a science career [29]. Interestingly, Kardash et al. suggested that the lack of interacting with people in the laboratory may be one factor that decreased students’ interest in science. One student was quoted as saying, “If there was any influence [of the research training], it is that I like research, but I would rather work with people” [29, p. 194]. Public engagement training has the potential to provide students with a different model for what a career in science can look like, one that might better align with their stated preferences.

### The current study

The goal of the current study was to gain a better understanding of the impact of public engagement training on undergraduate students. It investigated the impact of a unique summer program (described in more detail below) which provides cohorts of undergraduate students with an intensive experience that explicitly requires students to do public engagement training alongside their research training. Specifically, we wanted to answer the following questions:

- What are undergraduates’ perceived gains/challenges related to participating in informal public engagement training?
- How does public engagement training impact undergraduates’ attitudes about the importance of public engagement?
- What do undergraduates learn about science from participating in public engagement training?
- Do undergraduates continue to engage with the public after participating in our training program?

## Method

### Setting

The research and public engagement training took place in The Language Sciences Research Lab (or “The Language Pod”) at the Center of Science and Industry (COSI), a science center in Columbus, Ohio. Through a partnership between Ohio State University and COSI, the Language Pod operates out of the museum as part of a permanent exhibition. The lab has two primary goals: to conduct cutting-edge research across the language sciences and their related fields, and to connect with the public in the museum to educate visitors about language research and science as a whole [30]. To accomplish these goals, study participants are recruited directly from the floor of the museum to experience the scientific process themselves and contribute to ongoing research by associated faculty. The lab also performs a variety of interactive demonstrations with COSI visitors that highlight linguistic phenomena and promote the understanding of language as something that can be studied scientifically. In this way, both research and public engagement are important aspects of the lab’s day-to-day operations.

Since 2015, the Language Pod has hosted an intensive summer program, largely funded through an NSF REU (Research Experience for Undergraduates) Site grant. Students in the program participate in both the research and engagement activities in the Language Pod. They receive equal amounts of instruction focused on core issues related to research and informal science engagement. The topic of both the research and the engagement activities was linguistics and related language science fields (e.g., psychology of language, speech and hearing science). During the program, students take part in 30 hours of public engagement at the science museum by conducting language science demonstrations on the floor at the museum. In addition, students develop their own language science demonstration and present their original demonstrations at a final capstone event. Students are also assigned to a faculty-mentored research project. Their projects typically require them to collect data in the Language Pod and they present their research findings at the final capstone event.

The training in science engagement in the Pod is largely hands on. Students who are being trained are first shown the demonstrations by more experienced students. Then they have the opportunity to practice running the demonstrations on one another while receiving feedback from faculty and experienced students. Once students are approved to do so by faculty, they perform the demonstrations on the museum floor with visitors. While students are given tips by faculty about how to engage with different kinds of audiences, largely, students have to figure this out for themselves through trial-and-error.

### Participants

Twenty-four participants (19 women, 4 men, and 1 non-binary) took part in this study. Five of the participants were first generation college students. Nine students identified as white/Caucasian; 8 identified as Black/African American/Caribbean; 6 identified as Hispanic/Latina; and 1 identified as Chinese. These racial and gender categories were provided by the participants themselves. At the time of the interview, the REU program had run for five summers. All of the participants (70 in total) from these five cohorts were invited to participate in this study. The demographics of this sample are consistent with the demographics of the overall cohort. The language pod is interdisciplinary, thus, participants in this study came from several different backgrounds. The undergraduate majors of our participants were as follows: 6 Linguistics, 5 Psychology, 5 Speech and Hearing Science, 3 Language Arts, 3 Pre-Med, 1 Cognitive Science and 1 Business. At the time of the interviews, six participants had taken part 4.5 years earlier, five participants 3.5 years earlier, six participants were from 2.5 years earlier, three participants were from 1.5 years earlier, and four participants were drawn from the most recent summer program cohort (approximately 6 months before the interview). At the time of the interview, 6 were employed full time, 2 were still pursuing their undergraduate degree, 9 were students in graduate school, 3 were in post-baccalaureate positions, and 4 were job seeking. The initial recruitment email was sent on August 12, 2019 and the final interview was conducted on June 12, 2020.

Participants provided oral consent before being interviewed. This study and the use of oral consent was approved by the Office of Responsible Research Practices at Ohio State University, Columbus, OH. Oral consent was used so that participants identifying information (i.e., signatures) did not have to be stored.

### Procedure

We conducted a semi-structured, open-ended interview (see Table 1 for questions). After obtaining research ethics board approval both authors sent emails to all of the prior participants in the summer program describing the research and asking them to consent to a brief (less than 60 minute) interview about their experiences in the summer program. All of those who volunteered were interviewed. No follow-up requests were sent.

**Table 1:** Questions asked during interview.

Did you enjoy doing science outreach at the museum?
What do you feel you gained from participating in outreach?
What challenges did you face while doing outreach?
What was your most memorable experience doing outreach?
Did you feel like you got better at doing outreach over the summer? How did you know?
Did participation in the lab change how you think about scientists’ role in society?
Do you think it’s important for scientists to participate in outreach?
How did your views of science change after participating in our program?

Interviews were arranged by Zoom or in person, depending on the location of the interviewees. (After the onset of the COVID-19 pandemic, all interviews were conducted via Zoom.) The first author conducted the first three interviews with the assistance of a research assistant, but the remainder of the interviews were conducted by the research assistant alone. Our research assistant was not directly involved in any of the summer programs and like many of our former program participants, she was African-American. She was chosen to conduct the interviews so that the interviewees would feel comfortable discussing the full range of their experiences, including raising concerns they had with the program. Interviews ranged from 15 minutes to 45 minutes long and were recorded via Zoom and then transcribed verbatim by another research assistant. To ensure that confidentiality was not compromised each participant was assigned a code number and their interview transcript was stored digitally. A guide to interview numbers and institutions was kept separate from digital files; video-recordings were then destroyed.

### Coding

The video recordings of the interviews were transcribed verbatim. The initial transcripts were reviewed and every contentful utterance was identified. Statements that were unintelligible, repeated statements, and general discourse (e.g., “yeah,” “I guess”) were eliminated. Utterances were tagged for which question they were in response to.

In this article, we discuss a primary-level analysis of interview transcripts, viewing data critically and interpretively, using a form of emergent coding, in which major themes “emerge” from the participants’ responses [31]. The first step of the process was to code each utterance as to whether it was addressing one of our research questions; we identified five broad themes: Benefits, in which participants discussed the benefits of participating in the program; Challenges, in which participants discussed the challenges they faced engaging with the public; Importance of Public Engagement, in which participants discussed their views of public engagement more generally; Nature of Science, in which participants discussed how their views of science changed as a result of participation in our program; and Continuation, in which participants discussed their experiences with and reasons for continuing or not continuing to participate in public engagement.

Once these themes had been identified, every relevant utterance was coded for sub-themes within the broader theme by the first author. Those themes are presented below. The second author reviewed the codes to ensure agreement. All disagreements were resolved through conversation.

## Results

In what follows, we consider more specifically themes that emerged related to each of the research questions for this study. We note at the outset that our data is largely descriptive in nature. Given the exploratory nature of this study, we were primarily concerned with documenting the impressions of previous participants in the program. Note that although we prefer to use of the term “engagement” over “outreach”, in our interviews with participants we used “outreach” and our participants mirrored our use of that term. In this paper, we are using outreach and engagement interchangeably.

### RQ #1: What are undergraduates’ perceived gains from participating in public engagement training?

We asked participants to discuss what benefits they may have gained from engaging with the public. As Carpenter (2015) identified, three overarching themes related to gains emerged from participants’ discussions of their experiences: Participants reported academic, career, and personal gains as seen in Table 2.

**Table 2:** Undergraduates’ reported benefits from participating in public engagement training.

**Theme 1:** **Academic**		
**Code**	**Description**	**n**
Content	Engagement helped them learn about science concepts	8
Interest	Clarified academic interests	12
**Theme 2:** **Career**		
**Code**	**Description**	**n**
Options	Understanding career options	12
Engagement interest	Interest in engaging with the public in the future	8
Skills	Transferrable skills; communicating with different people	16
**Theme 3:** **Personal**		
**Code**	**Description**	**n**
Confidence	Feeling good about talking to new people	10
Fun	Expressed joy and enthusiasm about the demonstrations	11
Generate excitement	Sharing science and getting others excited	20
Group	Working together with the other members of the cohort	7
Improvement	Noticing that their communicating skills were improving	5
Science identification	Feeling a sense of science identity from doing demos	10

### Academic gains

Two themes emerged within the umbrella theme of academic gains. Participants discussed how public engagement training helped them better understand critical science content. Additionally, participants reported that the public engagement training helped them identify or clarify their own academic interests. We discuss each theme in more detail below.

#### Content

Eight participants mentioned that engaging with the public helped them either learn new content or gain a better understanding of concepts they had already learned about. All of these participants focused on the hands-on nature of engagement, As Participant 12 pointed out, the hands-on experience of learning “was different from what I was already learning in school.” Participant 17 pointed out that working in the science museum gave them the opportunity to “see what research looks like within the various subfields of linguistics.” Several participants mentioned that explaining concepts to others helped them learn the concepts. For example, Participant 5 said, “I had to learn it and learn it in a way so that I could explain it.” Similarly, Participant 13 said, “when you explain things to someone else, I feel like that’s when I understand things better.” This participant continued that in order to make things understandable to other people, “you have to think about the information that’s most relevant and most important for a certain population, which is something that in intro to psychology classes doesn’t really come up.” Participant 18 even noted that engaging with the public helped her gain confidence in an area she had previously struggled with, she said,

I know I was very terrified of phonology going into the program, just because I had had a bad experience with phonology in my intro to linguistics course. So, the little mini crash course on it, and then working with IPA and the ear model, that made me definitely more comfortable with it.

#### Interest

Twelve participants mentioned that the program influenced their academic interests. Nine of the participants mentioned that participating in the program increased their interest in pursuing graduate/professional training. Interestingly, three of these participants mentioned that engaging with the public influenced how they were thinking about their long-term goals. Participant 2 said, “[engaging with the public] was my favorite part… It really confirmed for me that I wanted to …maybe have a more applied approach to my interest in linguistics.” Participant 7 said that “the outreach was a very important part of it, because I really value that interaction with people and being able to share more now and making them curious.” Three participants indicated that participating in the program made them less interested in pursuing graduate training. They shared that this was because prior to participation they did not have much information about what graduate school and academic research entailed, and getting firsthand experience in academic research helped them decide it was not a good fit for them. All three valued learning this information earlier, rather than, as Participant 17 put it, “finding that out in my second year of grad school.” Interestingly, Participant 2, who decided that pursuing academic research was not for them, was applying for jobs in science education; she said, “I’m interested in science communication. I think participating in the program at least gave me the foundation for being able to do that.”

#### Career gains

There were three themes that emerged within the umbrella theme of career gains. Participants discussed how public engagement training helped them better understand different career options. Additionally, participants discussed a range of communication skills that they found to be useful in many work settings. Finally, participants reported an increased interested in engaging with the public as part of their long-term career goals. We discuss each theme in more detail below.

#### Options

Twelve participants mentioned that participating in the program helped them understand different career options. Some of the participants mentioned exposure to different disciplines and graduate programs that they had not heard of prior to participation. Five students specifically mentioned that public engagement was critical in helping them understand new options. Participant 3 said, “doing outreach helped me find a different part of myself.” Participant 4 said,

Outreach was my favorite part of working at the Pod. I liked it so much that after I finished the program, I looked for as many outreach opportunities I could do. Every single year after that, I was heavily involved with outreach. One of my dreams is to be able to create a medical laboratory outreach science program that’s like a summer week-long camp for kids. So that I can go to high schools and I can promote this major that I’m in and they can do this fun outreach program and learn about the major.

Similarly, Participant 20 actually went on to “start my own kind of center of outreach.” Two participants who were still planning to pursue academic research indicated that engaging with the public helped them think about their long-term goals. Participant 17 indicated that they were not interest in building a “lab only doing research, only conducting experiments” but wanted to incorporate, “education outreach and learning into the other activities I do.” Participant 3 said that “the informal science education I think was one of the best parts of it in terms of making you a better scientist. Just because I feel like the ability for scientists to talk the public is so important.”

#### Engagement interest

Eight participants indicated that participating in the program increased their interest in public engagement. Participant 4 said,

Before I did outreach, I didn’t really know that it could be so rewarding and I wanted it to be a part of my life. After I did that outreach, I realized that I wanted it to be a huge part of my life. With outreach, I felt extremely fulfilled and I feel like I was finally able to get a sense of direction with what I wanted to do with my life…when I realized I didn’t want to go to medical school, I was searching for what my true passions really were. I thought when I was the most happiest was when I was the most fulfilled and that’s when I was doing outreach [at COSI]. So it really gave me a sense of direction of what I wanted to do in the future, and that was to see if I could create some type of outreach program myself.

Participant 20 said,

I came into the program already loving science but what I knew was learning facts and classroom experiments. Being exposed to the idea of science outreach was something that completely changed my life. I found something I loved doing more than anything else I had encountered…A year after the summer program …I decided to pursue science education…I plan on making outreach an integral part of my career once I graduate and become a Medical Laboratory Scientist.

Importantly, the participants often reported being surprised by their interest in public engagement. For example, Participant 2 said, “When I applied, I remembered being really apprehensive about the outreach part of it and almost not applying because of it. Then, I got here and enjoyed it.” Although public engagement is a critical part of our training program, students who applied to our program were looking to gain research experience and viewed the public engagement training as an interesting bonus or something to be apprehensive about.

#### Skills

Sixteen participants reported that the public engagement training provided communication skills that would be helpful for them. Participant 22 mentioned that public engagement provided the “skills of knowing your audience and how to [do the demonstration] most effectively for them.” Participant 12 noted that after the engagement experience, “I can literally go out to anybody and talk to anybody about anything.” As Participant 1 put it, “[public engagement] was also like an entrepreneurial business skill: How to get someone excited about talking to you when they don’t know what you’re going to talk about.” Participant 14 mentioned that she put her public engagement training “on my resume and [talked] about that in interviews.”

### Personal gains

Five themes emerged related to personal gains from engaging with the public. Our participants enjoyed public engagement and discussed how it was inherently fun to do the activities, how they enjoyed generating excitement in others, and how much they enjoyed doing the activities with other members of their cohort. Participants also discussed how their engagement skills improved during their training. Additionally, participants discussed confidence gains as well as an increase in their sense of their own science identity.

### Confidence

Ten participants mentioned that one of the personal gains from public engagement was increased confidence. Some students mentioned that being on the floor of the science museum helped them feel “more professional,” while Participant 15 noted that being a “representative of OSU and also, of COSI” helped them gain confidence. Participant 2 noted that they gained the metacognitive skills of “assuring myself as I was explaining things that would be useful to other people” that she knew what she was talking about. Participant 3 noted that their confidence increased as a result of “experiment[ing] with different ways saying things to people and gauging how that affected their understanding of whatever demo we were doing at the time.” Finally, Participant 8 noted that the experience of engaging with the public was not a typical undergraduate experience, she said,

It was just really confidence-boosting to be an expert on the floor, doing things. Like as an undergraduate, you know, you’re constantly feeling like you don’t have enough information about things so feel a lack of self-confidence or stuff like that. So to be in a setting where it’s like, *I am the expert here; people are looking to me for information and they’re learning from me*, *and they’re super enthusiastic* was really a change.

### Fun

Eleven participants mentioned that public engagement was fun. Interestingly, participants often noted that they often didn’t expect to have fun. For example, Participant 3 said, “I didn’t expect to like it, and then I had a lot of fun doing it. I never saw myself as a teacher before and I feel like doing outreach helped me find a different part of myself.” Participant 23 mentioned that “I didn’t know how fun it was to show people [something about linguistics]… Now I can see why people are so into it… cause it’s fun!” Two participants mentioned that creating their own demonstration was particularly fun. Participant 24 said that it was “fun because I knew everything about that activity, and it was fun getting to interact with other people and demonstrating what I had created.”

#### Generate excitement

Twenty participants noted that generating excitement for museum visitors was the biggest reward for public engagement. Many of the participants focused on how rewarding it was that museum visitors were enthusiastic and curious about the language science demonstrations they were doing. For example, Participant 8 shared a story about her experience doing a demonstration with a preteen boy. After they were finished with the demonstration, he “brought back a horde of people” for her to do the demonstration with again. Participant 20, a Black woman noted, shared an even more poignant story. She explained that she was,

the only person of color on the floor for a second. And these two little girls that looked like me, they got to see me on the floor and they got to come up to me and ask me questions. So I really did enjoy my experience and that particular moment really made me, like, think, *Oh my god*, *this is really doing something*.

Participant 5 noted that it was rewarding to learn that even though “people might think of science as being boring or something that’s really difficult” it is possible to “accessible to everyone. It’s just how you present it.” Participant 17 shared a similar insight. She shared a story about doing a demonstration with an older man who was experiencing hearing loss. She shared that,

He wasn’t as entirely familiar with the anatomy of the ear as I had become over the course of the summer. And I wasn’t as familiar with his hearing issue. And so, it was really cool to kind of come to an understanding between the two of us. Him telling me about how his, how his hearing disorder came to be and how it affects his life, and then drawing connections to what we were actually physically seeing [because of the ear model she was using] in front of us. So, it was really cool to both learn and teach in that one interaction.

As Participant 7 put it, “I enjoyed the opportunity to be able to share science with the community, and I loved how you can make it interesting to people. And they can be either 5 years old or 70. You can still reach them.” Finally, three participants in particular noted that public engagement in linguistics was particularly rewarding because linguistics is not a commonly discussed science and many people are unfamiliar with it. Two participants in particular noted that engagement with children was exciting because the children would have the opportunity to “figure out that [linguistics] existed earlier.”

#### Group

Seven participants focused on the camaraderie of working with other program participants. For example, Participant 24 appreciated that although she was working independently, she “also had help” and that if she was not sure how to do a particular activity she could ask for “help from the other members of the cohort … or from the mentors.” Participant 17 reported enjoying “seeing my fellow members in the cohort grow over such a short period of time.” Participant 5 enjoyed “working with people who feel that same kind of passion.” Participant 3 said, “I remember the moments we shared on the floor, whether it was watching them do something or talking about a kid’s reaction.” Participant 3 continued by saying that they “enjoyed being in a group where I was able to learn new things, but also having conversations with everyone else who was going through the same experience.”

#### Improvement

When asked, all of the participants indicated that they saw their engagement skills improve over the course of the summer. Five participants specifically noted that watching their skills grow was particularly rewarding. For example, Participant 1 noted that they got excited when they did a new demonstration “for the first time and figuring out after a few times what worked best” and then transferring what they figured out from that first demonstration and trying out with “a brand new one.” Similarly, Participant 17 reflected on how important it was to

learn for myself what works in how to engage people and also what works in what various demographics of people [and what they] find engaging and most interesting. And that was, that was really cool to kind of learn how to tailor my engagement to do a variety of different types of people.

#### Science identification

Finally, ten participants described feeling a sense of science identification from engaging with the public. Science identity is an important construct that describes how someone thinks of themselves in relation to science. The construct was first described in the six strands of informal science learning set out in the National Research Council report “Learning Science in Informal Environments: People, Places, and Pursuits” [32], one of which was that learners in informal environments “think about themselves as science learners and develop an identity as someone who knows about, uses and sometime contributes to science. (p. 43)” Researchers in science education note the importance of developing a science identity because people who develop identities related to STEM engage with these topics more often and more deeply. A science identity, for example, increases the likelihood that students will, over the long term, continue to develop science literacy or even follow an educational pathway toward a science career or profession that requires or benefits from education or training in STEM [33]. In particular, participants reported that sharing their knowledge of science became important to them. For example, Participant 4 said, “I’m definitely a science person. But afterwards, I became even more of a science person because I wanted other people to love science as much as me.” Participant 5 similarly shared that she gets excited about language science and “learned that not everyone might share that same excitement due to lack of knowledge on the subject. Participating in the program allowed me to attempt to share that same excitement to the public, and I like to think that I rubbed off some of that enthusiasm to others!” Other participants were more specific about how rewarding it was to be a science role model for others. For example, Participant 12 shared that when working with children “we used to tell them that we were scientists” and in one instance after working with a child, he told her “that when he grew up, that he wanted to be a scientist.” Participant 23, a Black woman, shared an even more poignant story about having

three little Black girls [at the museum] see me and they just start smiling…, I guess it was either the mom or, like, the chaperone who said, “Yeah, they haven’t seen a Black girl up here doing the demos before, so, when they saw you, they were like, ’Oh my god, she’s here. She’s hanging out!” So I was like, “Oh my gosh, that’s dope.”

Participant 20, also a Black woman, had a similar experience of sharing exciting with a young Black girl. As that participant indicated, the experience

was more rewarding because it didn’t seem like I was, I guess, in a sense, assimilated. I had some dreadlocks in, they were in two big puffs. And I had the science coat on, and I was just there in all my Blackness. And they saw me. And I saw them see me. And it was, it wasn’t just like, *Oh*, *I’m looking at you*. It was like, *I’m looking at you*, *and I don’t think I’ve ever really seen you before*. So, for me, I think that was just the most memorable experience for me. And it, it really does touch my soul, and I think that’s why I’m so invested.

Similarly, participants often noted that their engagement experience shaped how they saw themselves as future scientists. For example, Participant 4 said, “I loved Outreach. It’s a big part of my life.” Participant 18 further explained, “I would really want to make sure that public outreach is a part of [being a scientist]. Because I don’t want my research to just end up growing dusty on a bookshelf.”

To summarize, participants found science engagement training during their undergraduate studies to be highly beneficial. In terms of academic gains, our participants noted that science engagement helped them learn scientific content and clarified their academic interests. They also noted that science engagement training helped them understand different future career options, increased their interest in careers with a public facing component, and improved skills, such as communicating with different kinds of people, that are useful in the workforce. Finally, participants said that engaging with the public was fun and increased their confidence in their own skills in ways that they did not get in their regular academic studies. Participants enjoyed the opportunity to share their enthusiasm about science with others and working with and learning from other members of the cohort. In particular, participants noted that public engagement gave them several opportunities to notice that their skills were improving and increased their own identification with science.

### RQ #2: What are undergraduates’ perceived challenges faced when participating in informal public engagement training?

We asked participants to describe the challenges they faced engaging with the public while participating in our program. As indicated in Table 3, participants described two overarching themes related to engagement during the program, there were challenges due to the nature of engagement and personal challenges.

**Table 3:** Undergraduates reported challenges related to informal public engagement training.

**Theme 1:** **Working on the Museum Floor**		
**Code**	**Description**	**n**
Children	No prior experience working with children	4
Flexibility	Needing to change presentation based on museum visitor needs	5
People losing interest	Keeping museum visitors engaged and interested	7
**Theme 2:** **Personal**		
**Code**	**Description**	**n**
Cultural barriers	Not being familiar with some culturally specific material in the demos or being an ESL speaker	2
Anxiety	Feeling uncomfortable approaching strangers in an informal and unstructured environment; no prior experience with public engagement	14

### Working on the museum floor

When discussing the challenges undergraduates faced doing informal science engagement, three themes emerged that were related to the nature of working with people at a science center. Specifically, participants noted a lack of prior experience working with children, the need to be flexible to meet the demands of many different kinds of people who visit the science museum, and people losing interest in the demonstration for a variety of reasons.

#### Children

Although many people visit science centers, children are often the most likely to engage with science demonstrations. Four of our participants indicated that they did not have prior experience working with children and found doing so initially challenging. As Participant 19 put it, “Even though I do love children, there is something very intimidating about holding their attention for an extended period of time.”

#### Flexibility

Five participants discussed the challenge of finding different ways of explaining the science content to different kinds of people. For example, Participant 16 discussed the challenge of taking complex ideas and simplifying them in a way that was both “accurate” and “accessible.” Other participants focused on the pressure to do this in real time. For example, Participant 8 discussed the challenge of being in the “moment” and trying to I had to “make this concept more understandable for this person.” Participant 1 shared a similar sentiment saying that “the big challenge was having to be able to think on your feet. If you’re talking to someone and they’re not interested in what you’re showing them, how do you pivot?” However, this participant realized that developing this skill was “a good challenge.”

#### People losing interest

One of the advantages of informal learning is that participants have the power to choose what they learn about at any given moment. Seven participants in this study found that to be challenging. As Participant 9 pointed out, museum visitors are “not obligated to talk to you at all so they may just walk away in the middle of your conversation. So, that was a challenge, you always had to make sure that you were engaging.”

### Personal

Two themes emerged that were more related to undergraduates’ individual differences rather than the nature of public engagement. Participants discussed some cultural barriers involved with interacting with the content in the demonstration or working with people in the museum and anxiety around working in a new and unfamiliar environment.

#### Cultural barriers

Two participants mentioned specific cultural barriers to engagement. Participant 7 was not a Native speaker of English, and she shared that “English isn’t my first language, sometimes I would forget words or like stuff like that in the moment, so…sometimes it would be like a gap in my speech…while I waited to remind myself of the word, but…it’s just part of the process, so it wasn’t like a huge problem.” While Participant 24 noted being unfamiliar with some of the culturally specific content in some of the demonstrations.

#### Anxiety

Several participants noted being anxious engaging with the public either because it was a brand new experience or more specifically that they were nervous about interacting with strangers in the science museum. Four participants noted that at the beginning, they did not feel like they were good at doing the demonstrations, given that they had no prior experience engaging with the public or teaching of any kind. However, all four of them recognized that with practice they got better. As Participant 22 put it, “I’d do an outreach session and be like, *Man*, *I really should’ve done that better*, so, just going back and thinking, how could I do this more effectively.” Ten participants specified that they were initially nervous about interacting with people who were unfamiliar. However, all of the participants noted that with practice, they overcame this challenge. For example, Participant 6 said, “I consider myself to be like a kind of shy person, but like at first when I wasn’t used to it, it’s like "oh my god, I really have to go talk to these people." But like, after like a few times, you get used to it.” Similarly, Participant 12 shared, “The only thing that I had trouble with at first was just getting the courage to go up to people. But after like I had practice with it, then it was nothing.”

To summarize, participants did not find working with the public to be too difficult, with their biggest challenge being anxiety due to the fact that they were learning how to do this work for the first time. Participants also found that initially it was challenging to figure out how to work with children, how to keep people’s interest, or how to respond to different participants’ needs, but all of them agreed that they got better at all of this with practice. Finally, two participants noted cultural barriers, either from not using English as their primary language or not knowing some of the culturally specific content in the demonstrations.

### RQ #3: How does public engagement training impact undergraduates’ attitudes about the importance of public engagement?

Again, one of our hypotheses was that early training in public engagement would impact scientists’ attitudes about public engagement. During the interview, we asked participants whether they thought public engagement was important. Twenty-one participants agreed that public engagement is important. Of those participants, two qualified their “yes” response suggesting that for some areas of science public engagement may be more important. (Note: These two participants did not elaborate on what areas of science they thought should be most likely to engage with the public about science.) Fourteen participants elaborated on why they feel public engagement is an important piece of being a scientist. As Table 4 indicates, three themes emerged from their elaborations.

**Table 4:** Undergraduates’ views on why scientists should be engaged with the public.

Code	Description	n
Access	Public has a right to the knowledge scientists generate; public would benefit from better understanding of science	10
Interest	Scientists have a duty to encourage others to pursue science	2
Trust	Engagement efforts build trust with the public	2

#### Access

The majority of participants indicated that the public has a fundamental right to the knowledge that scientists generate, and that people could make better informed decisions if they understand scientific findings. For example, Participant 1 noted that, “one of the many problems with the academy is that all of the information stays inside and people don’t have access to it.” This participant further elaborated that people have a right to “learn [about science] in an accessible way” and that teaching people about their science “should be a priority for scientists.” Participant 2 said that “if people have the information, they can make better decisions about what to do in the future and how to apply science to their own lives. I think that’s really important, and it starts in childhood and also starts with making science fun and accessible and engaging.” Participant 22 noted that sometimes “people really struggle to understand what’s being communicated to them by a scientist” because scientists rely on “jargon.” Thus, this participant took away that scientists should have some practice explaining concepts without using [jargon].” Finally, Participant 23 learned that for her public engagement was an important part of doing science because she feels that “other people [must] understand what my work is to give it value.”

#### Interest

Two participants indicated that part of a scientist’s job is to encourage others to pursue science. For example, Participant 4 said “getting people to love science is just one of the small parts of being a scientist” while Participant 20 said that “it’s really important to just show these kids what you can do within this discipline and how cool it can be” and further expanded that scientists should do this kind of public engagement because “it would be very beneficial to society.”

#### Trust

Two participants noted that some people without scientific training do not necessarily trust science or scientists. These participants noted that public engagement could help people “understand what’s going on, and not fear [scientists], and not have this taboo against science” (Participant 7) and that public engagement can bridge “the gap between the scientists and the rest of society” (Participant 9). Importantly, science engagement has been shown to be one way that scientists can help build trust with the public [11]. While this is not something that was directly communicated in our program, these two students nevertheless recognized this impact of science engagement after participating.

To summarize, all of our participants agreed that scientists should engage with the public. Most of the participants argued that the public have a fundamental right to the knowledge that scientists generate and that their lives would be improved with more access to that knowledge. Two participants noted that scientists have a duty to encourage others to pursue their field of study. And finally, two participants noted that scientists should engage with the public in order to increase trust in the scientific process.

### RQ #4: How does training in this setting impact undergraduates’ understanding of science?

Finally, we wanted to understand how participating in our program impacted participants’ understanding of science. The traditional model of science focuses on developing a hypothesis, designing an experiment to test the hypothesis, interpreting the data, and then repeating the process. Importantly, the traditional model often focuses on the singular scientist, working alone in a laboratory and does not include engagement with the public as a core part of “doing science” [34,35]. Traditional models of science often emphasize the importance of science aptitude and imagine that only some people are suited for careers in science [36]. We were interested in whether experience with public engagement might shape how undergraduates view science more broadly.

Three themes emerged from participants’ responses as shown in Table 5. The first, nature of science, was more related to participants’ research experiences. However, two themes emerged related to how public engagement training impacted undergraduates’ understanding of science.

**Table 5:** Undergraduates understanding of science after public engagement training.

Code	Description	n
Nature of science	The scientific method is a process and messier than previously understood	11
Engaged	Scientists should be engaged with the public because science should be accessible and scientists have a duty to inform the public	13
Science is for everyone!	Everyone can get excited about science; there’s a need for diversity among scientists	10

#### Nature of science

Unsurprisingly, many students mentioned learning a lot about the nature of science because of participating in our program. For example, Participant 1 mentioned learning that there is “no such thing as 100% accuracy in research and science… [because] there are so many variables that are so hard to control for.” Participant 4 shared that “seeing the [research] process from start to finish was incredible. Science became tangible and not just an assortment of lecture notes I have to learn each semester. I realized how much work goes into answering a research question and how much paperwork and planning is involved. It wasn’t just about knowing science but knowing how to organize people.” Finally, Participant 9 noted that it was important to understand that science involves a lot of “trial and error. And I think making sure that you’re not getting discouraged and understanding that it’s a part of the process.”

#### Engaged

Thirteen participants responded that the public engagement training also impacted how they think about science. These participants were surprised that science could engage the public as their previous experiences with science hadn’t taught them this. For example, one participant (18) said, “I feel like a lot of times especially in academia, all this research can feel like it’s in a bubble.” Another participant (10) indicated that they learned that “science can be more than [animal testing and test tubes], y’know. Scientists can work in a museum and teach people things through fun.” Another participant (20) shared that

[I] always saw scientists that were just, like, mixing chemicals. And just seeing the lab [at COSI] itself … it was just a very different look than what I was used to, so it gave me a definitely a different perspective on what a lab *can* look like, not what it’s supposed to look like.

Another participant (19) shared that her previous ideas about what science labs looked like made her feel like “research is kind of boring.” But she went on to say that the public engagement made research “really fun. I feel like writing the stuff up was the, the boring part. But, like, the actual part of, like, doing the research, that was fun. And it made me think differently [about what a lab can look like]. You don’t have to be just being stuck in a lab by yourself isolated from everybody.” Some of the participants further expanded that learning about public-facing science made them interested in continuing to engage with the public in their future science careers. For example, one participant (16) shared that she had never considered the fact that scientists could be “involved in society and do research that matters.” That same participant went on to say that discovering that public-engagement could be part of being a scientist became “a huge part of the way I envision my career and my own entrance into academia, both as, as sort of an engaged [scientist] whose research is impactful to the people who I work with, collaborative research, community based questions, and then making sure that the important part of that research also gets communicated to publics beyond academia and beyond the group that I work with.” Another participant (15) shared that “after that summer, I realized that I wanted to be in a position where I could be closer to conversations around application and impact.” Finally, one participant (8) shared they their experience taught them that public engagement should be a component of the scientific process and that “linguists should be more involved in some of the conversations that are being had [in the public].”

#### Science is for everyone!

Ten participants shared that their experience with the program instilled in them the value that diversity in science is important. Some of the participants focused on the importance of engaging everyone in science whether or not they are interested in pursuing science. For example, one participant (6) said that although “a lot of people might think of science as being boring or something that’s really difficult,” it was important to realize that if you “break it down into really, really simple ideas for kids and families to understand, you realize it’s accessible to everyone.” Another participant (9) similarly shared that,

[public engagement] showed me that anyone can *do* science. In fact, it seems like all of us have a little bit of an innate ability to be a scientist by finding our way through life by trial and error. I understand now that science isn’t reserved for the elite who wear white lab coats. Science is for every one of us who aims to gather a greater understanding of the world around us, and we achieve that understanding through science.

Other participants shared the importance of having diversity in terms of who is doing science. For example, Participant 20 who shared the story above about working with three Black girls in the museum shared that she “learned that seeing people like you in places that you wouldn’t expect them to be is very, very important. Kids need that.” Participant 23 shared a similar sentiment saying that she realized that she “can be like a catalyst for them to [do] science. Cause they were like, *Oh yeah*, *we can hang out in the museum*. But now it’s, like, *Someone that looks like me doing science*”

In summary, participants in this program reported learning that science is an iterative process, full of starts and stops, and that having an experiment fail is normal and should not be a discouragement. In addition, they reported a strong sense that scientists have a responsibility to engage with the public. Finally, they also discussed the role science engagement can play in generating interest in science in children and noted the role engagement may play in diversifying who participates in science.

### RQ #5: Do participants continue to engage with the public?

Finally, we wanted to know whether participants in our program continued engaging with the public after leaving our program. Eleven participants mentioned not having opportunities to engage with the public after our summer program. For example, Participant 24 noted that she could not find “a lot of like programs, at least in my college” while Participant 8 noted that “the Pod is a very special kind of place.” However, Participants 17, who was unable to find opportunities, did mention that she had reached out to a faculty member at her home undergraduate institution “about kind of doing something similar at our local science museum. I talked to her about some of the learnings I’d had over the summer.”

Importantly, nine participants mentioned finding opportunities to participate in public engagement. Most of these involved joining us where we participated in the Family Science Days event at the American Association for the Advancement of Sciences (AAAS) conference. However, importantly three of our participants used their experience in our program to create their own science engagement opportunities. Participant 5 was working in a laboratory that recruited participants from outside the university, which meant they often had tables set up at public events. This participant used some of the demonstrations they learned in our lab to set up a small engagement component for their research recruitment events. Participant 21 discussed creating an engagement program designed to teach young children about the field of speech and hearing science. Finally, Participant 9 used some of the demonstrations they learned in our lab to do engagement with college freshmen to help them learn about research opportunities in psychology.

In sum, although our participants were interested in science engagement, most of them did not find additional opportunities for science engagement work outside of participating in our program. However, impressively three students went on to create their own engagement projects at their home institutions.

## Discussion

The goal of this study was to explore the impact of public engagement training on undergraduates. We interviewed 24 participants who had taken part in a summer program dedicated to both science and public engagement training to better understand how public engagement training impacted their experiences as scientists in training. In particular, we wanted to understand undergraduates’ perceived gains and challenges related to public engagement, and also how public engagement training influenced their thoughts about public engagement, the scientific process, and their own identities as scientists.

Importantly, these data are descriptive in nature and only represent the views of a small number of (mostly women) participants. In addition, the science topic that the students focused on in their engagement was language, which is relatively easy to get people to engage with as everyone uses language every day. In our previous work, we found that people have very fond memories related to language [37] and this may have increased the likelihood that the participants in this study had such a positive experience. However, despite these caveats, these data suggest that undergraduate students benefit from incorporating training in public science engagement into their scientific education.

The participants in this study noted several important academic, career, and personal benefits, similar to the gains reported by Carpenter (2015). In terms of academic gains, our participants noted that explaining concepts to museum visitors helped them understand the science content better themselves and helped clarify their own academic interests. In terms of career gains, our participants shared that public engagement helped them better understand future career options, it got them interested in public engagement in the future, and that it improved their communication skills. Finally, in terms of personal gains, participants noted that public engagement was fun, confidence-boosting, they enjoyed generating excitement in others and working with other members of the cohort, it gave them a chance to see their skills improve, and it increased their own science identities.

Most participants did not find public engagement challenging while working in our lab, and we note that all of the participants saw the occasional challenges as opportunities. They all noted that they got less anxious over time and that their ability to keep people’s interest and make the content accessible, even to children, improved. When participants were discussing the gains and challenges related to public engagement, "improvement" was an important theme that came up during the conversations. One important reason why public engagement is important for undergraduate students is that it gives them many opportunities to demonstrate their own expertise. As one of our participants mentioned, it is rare for undergraduate students to feel that they have expertise in anything. Even when students get involved in research, much of their experience is learning new concepts and ways to work, and generally feeling concerned about their ability to do things correctly. Public engagement, especially in a museum setting, gives students the opportunity to take more ownership over their work. Although we provide students with guidelines for doing the demonstrations and give them ample time to practice before going out onto the museum floor, students must figure out for themselves how best to present the information. One helpful aspect of the setting is that students get immediate feedback from museum visitors about how well they are doing. Our participants often noted how they valued the feedback from museum visitors and were able to use it to hone their engagement skills.

Importantly, our participants indicated that the public engagement experience was useful regardless of whether or not they continued in academia. They valued the communication skills they gained, in particular, and the experience of practicing designing their message based on their audience’s needs. Our participants noted that this was not a skill that traditional academic settings allowed them to hone and they felt this ability was useful in many settings.

In addition to understanding their perceived benefits, we wanted to understand how training in public engagement influenced our students’ attitudes about publicly engaged science. One way to get more scientists engaged in public engagement may be to begin training them early to doing this kind of work. Our data suggest that this might be an effective way to shift more scientists into public engagement. First, all of our participants agreed that public engagement is an important part of a scientist’s duties. The participants who provided rationale suggested that engagement was important because the public has a right to any knowledge that scientists generate. Of course, we did not measure whether this attitude shifted as a result of our program, as we did not ask students about this prior to participation. Also, our program is advertised as providing public engagement training, so presumably only students who are interested in, or at best, not turned off by the idea of public engagement training apply to our program. That said, we can be confident that participating in public engagement did not cause our students to lose interest in engagement. Importantly, the public engagement training did not take away from students’ research training. Several participants noted that public engagement helped them better understand some of the academic content and helped them clarify their own research interests.

In addition, all of our participants noted that they got better at engaging with the public during the course of our program. This is important because many faculty report not doing public engagement because they feel they aren’t skilled at it [6,8,9]. Training scientists early on may build their confidence in their ability to do that work later. In fact, three of our participants noted that they took their experiences to build new engagement opportunities in their home communities and one participant discussed this possibility with a professor at their home institution. This strongly suggests that the early introduction of public engagement training is highly impactful and can lead to future engagement.

Perhaps most importantly, our data also suggests an additional benefit of incorporating public engagement into undergraduate science training, namely, that it may be an important way to broaden participation in science. As previously mentioned, Kardash et al. suggested that one reason why students lose interest in pursuing a career in science is that they perceive a science career to consist of locking oneself away in a laboratory and not engage with other people. Understandably, many students do not view this as an attractive long-term career option. In fact, several of our participants expressed similar ideas, one in particular noted not wanting to be locked away in an “ivory tower.” However, participating in public engagement gave our participants a different view of what a career in science can look like. Our participants noted that interacting with other people, whether it was generating interest in museum visitors or working together with other members of the cohort, to be one of the most important parts of their public engagement experience. Moreover, the positive impact of engagement on career goals was particularly striking among our students from under-represented minorities. Among the participants who indicated that the program had increased their interest in engagement and increased their self-identity as a scientist, the majority (75% and 80% respectively) were African-American or Latina students.

A lack of confidence in public engagement skills is only one barrier to engaging with the public often reported by faculty. Many faculty report structural barriers to public engagement. As one of the faculty member participants in Andrews et al. (2005) noted “You’ve only got a certain amount of time, so if we’re spending a lot of time at K-12, it means we’re not putting that time into the undergraduates and the graduates-I don’t think people are honest about this particular issue, because if you’re going to put a lot of effort into outreach then someone is going to suffer. Either your own research is going to suffer, or your undergraduate teaching is going to suffer, or your graduate program is going to suffer. Assuming that you’re not going to start working longer hours.” [12, p.286]. We believe that our data suggests that faculty do not have to sacrifice undergraduate and graduate students training in order to engage with the public. One solution is to integrate public engagement training as part of teaching and research supervision duties. As our data suggests, this allows faculty to participate in public engagement while also providing students with a rich and clearly beneficial training experience. Faculty then get the opportunity to engage with the public without taking away from their students’ education.

However, we also must agree with the faculty member quoted above that there is not enough institutional support for faculty to engage with the public even when incorporating student training into that work. Building and maintaining relationships with community partners takes time and resources which faculty do not always have. In addition, this work is not always valued in the same way as more formal means of knowledge sharing (e.g., publishing journal articles). Providing more institutional support for faculty to lead student-focused public engagement may be an important way to achieve important outcomes, namely encouraging more diverse students to pursue careers in science and increasing the public’s access to scientific knowledge.

## References

[1] O’Meara K, Jaeger AJ. Preparing future faculty for community engagement: Barriers, facilitators, models, and recommendations. Journal of Higher Education Outreach and Engagement. 2016; 20: 127–50.

[2] National Science Foundation. Merit review broader impacts criterion: representative activities. National Science Foundation. 2007; 11: http://www.nsf.gove/pubs/gpg/broaderimpacts.pdf.

[3] Greenwood MRC, Riordan DG. Civic scientist/civic duty. Science Communication. 2001; 23: 28–40.

[4] Leshner AI. Public engagement with science. Science. 2003; 299: 977–977. doi: 10.1126/science.299.5609.977 12586907

[5] Brownell SE, Price JV, Steinman L. Science communication to the general public: why we need to teach undergraduate and graduate students this skill as part of their formal scientific training. Journal of Undergraduate Neuroscience Education. 2013; 12: E6–E10. . 24319399 PMC3852879

[6] Ecklund EH, James SA, Lincoln AE. How academic biologists and physicists view science outreach. PloS one. 2012; 7: e36240. doi: 10.1371/journal.pone.0036240 22590526 PMC3348938

[7] Mathews DJH, Kalfoglou A, Hudson K. Geneticists’ views on science policy formation and public outreach. American Journal of Medical Genetics. 2005; 137: 161–169. doi: 10.1002/ajmg.a.30849 16082707

[8] Poliakoffl E, Webb TL. What factors predict scientists’ intentions to participate in public engagement of science activities? Science Communication. 2007; 29: 242–263. doi: 10.1177/1075547007308009

[9] Shanley P, Lopez C. Out of the loop: why research rarely reaches policy makers and the public and what can be done. Biotropica. 2009; 41: 535–544. doi: 10.1111/j.1744-7429.2009.00561.x

[10] Carpenter SL. Undergraduates’ perceived gains and ideas about teaching and learning science from participating in science education outreach programs. Journal of Higher Education Outreach and Engagement. 2015; 19: 113–146.

[11] Boyette T, Ramsey J R. Does the messenger matter? Studying the impacts of scientists and engineers interacting with public audiences at science festival events. Journal of Science Communication. 2019; 18: A02. doi: 10.22323/2.18020202

[12] Andrews E, Weaver A, Hanley D, Shamatha J, Melton G. Scientists and public outreach: Participation, motivations, and impediments. Journal of Geoscience Education. 2005; 53: 281–293. doi: 10.5408/1089-9995-53.3.281

[13] deKoven A, Trumbull DJ. Science graduate students doing science outreach: Participation effects and perceived barriers to participation. Electronic Journal of Science Education. 2002; 7.

[14] Laursen S, Liston C, Thiry H, Graf J. What good is a scientist in the classroom? Participant outcomes and program design features for a short-duration science outreach intervention in K-12 classrooms. CBE—Life Sciences Education. 2007; 6: 49–64. doi: 10.1187/cbe.06-05-0165 17339394 PMC1810206

[15] Page M, Wilhelm MS, Regens N. Preparing Graduate Students for Teaching: Expected and Unexpected Outcomes From Participation in a GK—12 Classroom Fellowship. Journal of College Science Teaching. 2011; 40:32–37.

[16] Stamp N, O’Brien T. GK—12 partnership: A model to advance change in science education. BioScience. 2005; 55: 70–77.

[17] Laursen S, Thiry H, Liston C. The impact of a university-based school science outreach program on graduate student participants’ career paths and professional socialization. Journal of Higher Education Outreach and Engagement. 2012; 16: 47–75.

[18] Thompson SL, Collins A, Metzgar V, Joeston MD, Shepherd V. Exploring graduate-level scientists’ participation in a sustained K-12 teaching collaboration. School Science and Mathematics. 2002; 102: 254–265. doi: 10.1111/j.1949-8594.2002.tb17884.x

[19] Moskal BM, Skokan C, Kosbar L, Dean A, Westland C, Barker H, et al. K‐12 outreach: Identifying the broader impacts of four outreach projects. Journal of Engineering Education. 2007; 96: 173–89. doi: 10.1002/j.2168-9830.2007.tb00928.x

[20] Lopatto D. The essential features of undergraduate research. Council on Undergraduate Research Quarterly. 2003; 24: 139–142.

[21] Holland B. Factors and strategies that influence faculty involvement in public service. Journal of Public Service & Outreach. 1999; 4: 37–43.

[22] Johnson WB, Behling LL, Miller P, Vandermaas-Peeler M. Undergraduate research mentoring: Obstacles and opportunities. Mentoring & Tutoring: Partnership in Learning. 2015; 23: 441–453. doi: 10.1080/13611267.2015.1126167

[23] Wayment HA, Dickson KL. Increasing student participation in undergraduate research benefits students, faculty, and department. Teaching of Psychology. 2008; 35: 194–197. doi: 10.1080/00986280802189213

[24] Landrum RE, Nelsen LR. The undergraduate research assistantship: An analysis of the benefits. Teaching of Psychology. 2002; 29:15–19.

[25] Ishiyama J. Does early participation in undergraduate research benefit social science and humanities students? College Student Journal. 2002; 36: 381–387.

[26] Kierniesky NC. Undergraduate research in small psychology departments: Two decades later. Teaching of Psychology. 2005; 32: 84–90.

[27] Kolb DA. Experiential learning: Experience as the source of learning and development. New Jersey: Prentice-Hall. 1984.

[28] Walker R, Clary RM, Wissehr C. Embedding sustainability instruction across content areas: Best classroom practices from informal environmental education. Journal of Geoscience Education. 2017; 65:185–193. doi: 10.5408/16-167.1

[29] Kardash CM Wallace M, Blockus L. Undergraduate research experiences: Male and female interns’ perceptions of gains, disappointments, and self-efficacy. Creating effective undergraduate research programs in science: The transformation from student to scientist, 2008; 191–205.

[30] Wagner L, Speer SR, Moore LC, McCullough EA, Ito K, Clopper CG, Campbell-Kibler K. Linguistics in a science museum: Integrating research, teaching, and outreach in a language sciences research lab. Language and Linguistics Compass. 2015; 9: 420–431. doi: 10.1111/lnc3.12164

[31] Glaser BG, Holton J. Remodeling grounded theory. In Forum qualitative sozialforschung/forum: qualitative social research. 2004.

[32] Bell P, Lewenstein B, Shouse AW, Feder MA. editors. Learning Science in Informal Environments: People, Places, and Pursuits. Washington D. C.: The National Academies Press. 2009.

[33] Dorph R, Cannady MA, Schunn CD. How science learning activation enables success for youth in science learning experiences. The Electronic Journal for Research in Science & Mathematics Education. 2016; 20: 8.

[34] Diekman AB, Brown ER, Johnston AM, Clark EK. Seeking congruity between goals and roles: A new look at why women opt out of science, technology, engineering, and mathematics careers. Psychological Science. 2010; 21: 1051–1057. doi: 10.1177/0956797610377342 20631322

[35] Diekman AB, Clark EK, Johnston AM, Brown ER, Steinberg M. Malleability in communal goals and beliefs influences attraction to STEM careers. Journal of Personality and Social Psychology. 2011; 101: 902–918. doi: 10.1037/a0025199 21859224

[36] Osborne J, Simon S, Collins S. Attitudes towards science: A review of the literature and its implications. International Journal of Science Education. 2003; 25: 1049–1079. doi: 10.1080/0950069032000032199

[37] Wagner L, Patson ND, Awani S. What does the public think about language science?. Language. 2022; 98: e224–e249. doi: 10.1353/lan.2022.0029

